# Targeted sequencing of the 9p21.3 region reveals association with reduced disease risks in Ashkenazi Jewish centenarians

**DOI:** 10.1111/acel.13962

**Published:** 2023-08-22

**Authors:** Yizhou Zhu, Seungjin Ryu, Archana Tare, Nir Barzilai, Gil Atzmon, Yousin Suh

**Affiliations:** ^1^ Department of Obstetrics and Gynecology Columbia University New York City New York USA; ^2^ Department of Pharmacology, College of Medicine Hallym University Chuncheon Gangwon Korea; ^3^ Department of Genetics Albert Einstein College of Medicine Bronx New York USA; ^4^ Institute for Aging Research Albert Einstein College of Medicine Bronx New York USA; ^5^ Department of Medicine Albert Einstein College of Medicine Bronx New York USA; ^6^ Department of Human Biology, Faculty of Natural Sciences University of Haifa Haifa Israel; ^7^ Department of Genetics and Development Columbia University New York City New York USA

**Keywords:** 9p21, age‐related disease, aging, centenarians, longevity, population genomics

## Abstract

Genome‐wide association studies (GWAS) have pinpointed the chromosomal locus 9p21.3 as a genetic hotspot for various age‐related disorders. Common genetic variants in this locus are linked to multiple traits, including coronary artery diseases, cancers, and diabetes. Centenarians are known for their reduced risk and delayed onset of these conditions. To investigate whether this evasion of disease risks involves diminished genetic risks in the 9p21.3 locus, we sequenced this region in an Ashkenazi Jewish centenarian cohort (centenarians: *n* = 450, healthy controls: *n* = 500). Risk alleles associated with cancers, glaucoma, CAD, and T2D showed a significant depletion in centenarians. Furthermore, the risk and non‐risk genotypes are linked to two distinct low‐frequency variant profiles, enriched in controls and centenarians, respectively. Our findings provide evidence that the extreme longevity cohort is associated with collectively lower risks of multiple age‐related diseases in the 9p21.3 locus.

## Graphical abstract

In this comprehensive sequencing analysis, we investigated the relationship between the 9p21.3 genetic hotspot and extreme longevity in Ashkenazi Jewish centenarians. Our findings revealed a significant depletion of risk alleles associated with age‐related diseases in this locus among the centenarians. This suggests that extreme longevity may involve a collectively lower genetic risk of multiple age‐related diseases in the 9p21.3 locus.

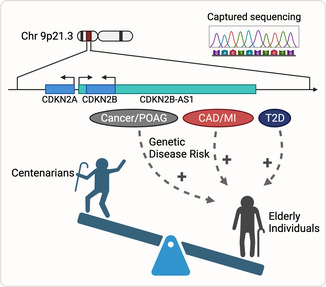

**Abbreviations**
AJ: Ashkenazi Jewish
CVD: cardiovascular disease
GWAS: genome‐wide association studies
LD: linkage disequilibrium
MAF: minor allele frequency
PCA: principal component analysis
POAG: primary open angle glaucoma
SKAT: sequence kernal association test
SNP: single nucleotide polymorphism
SVC: support vector classifier
T2D: type 2 diabetes

Longevity is a multifaceted phenotype influenced by a combination of environmental and genetic factors. Twin studies have demonstrated that longevity is moderately heritable (estimated at 20%–30%), with genetic factors playing a more significant role in achieving extended longevity at higher ages (Hjelmborg et al., 2006). Extremely long‐lived individuals often exhibit healthy aging characteristics, such as the absence or delayed onset of age‐related diseases, suggesting that they may be genetically protected from age‐related disease risks (Perls, 2006). However, previous research has shown that disease risk alleles identified through genome‐wide association studies (GWAS) are commonly found in longevity cohorts (Brooks‐Wilson, 2013). This implies the existence of alternative mechanisms for controlling these disease risks, such as the presence of protective rare variants.

The 9p21.3 non‐coding locus, located upstream of the INK4/ARF (CDKN2A/B) genes, remains one of the most consistently replicated GWAS hotspots (Hannou et al., 2015; Jeck et al., 2012). This locus has been linked to risk of multiple age‐related diseases, including cardiovascular diseases (CVD), Type 2 diabetes (T2D), glaucoma, and multiple cancers (Cugino et al., 2012; Helgadottir et al., 2008; Rahmioglu et al., 2014; Samani et al., 2007; Sherborne et al., 2010; Wiggs et al., 2012; Wrensch et al., 2009). In contrast to the strong association of 9p21.3 with age‐related diseases, fewer studies have explored the locus's relationship with longevity. A genome‐wide association study of the New England Centenarian cohort reported a weak association signal for rs1063192, a 3′ UTR variant on CDKN2B, which has also been linked to glaucoma (Kotake et al., 2011). The UK Biobank identified an association between rs1556516, a CAD‐related variant, and parental longevity (Pilling et al., 2017). Another top variant associated with coronary artery disease, rs1333049, was found to be connected to longevity in a Spanish centenarian cohort as well as the Wellderly healthy aging cohort (Erikson et al., 2016; Pinos et al., 2014). However, this association was not confirmed in two Japanese studies performed by independent groups (Congrains et al., 2015; Pinos et al., 2014). It is important to note that the majority of studies have focused on genotyping common disease variants. Consequently, the implications of rare variants in this region with respect to longevity remain largely uncharacterized.

To comprehensively investigate the association between extreme longevity and the 9p21.3 genotype, we conducted a sequencing study of this locus in Ashkenazi Jewish (AJ) centenarians (*n* = 450; mean age = 98 for cases and *n* = 500; mean age = 73 for controls). The AJ population is genetically homogenous (Ryu et al., 2016; Shlush et al., 2008). Utilizing pooled capture sequencing, we sequenced the 230 kb GWAS interval (chr9: 21,950,000‐22,180,000, hg19) with an average 30× depth (Ryu et al., 2018). To validate sequencing results, we genotyped 32 SNPs showing significant allele frequency difference between the two groups (Table S1). The result was highly consistent between the methods (*r* ^2^ ≥ 0.99, Figure S1).

We identified 2216 variants, including 2056 single‐nucleotide polymorphisms (SNPs) and 160 indels (Table 1a, Table S2). Comparing these variants with the current SNP database (SNP149) revealed that 785 out of 2216 (35.4%) were novel, comprising 664 SNPs and 47 indels. Among all novel variants, 95% (743) were rare (minor allele frequency <1%), and 78% (616) were singletons.

**TABLE 1:** (A) Summary of variants identified by sequencing of 9p21.3 in 450 centenarians and 500 controls arranged by minor allele frequency ranges. Variant annotation was performed with dbSNP database 149. (B) Functional annotation of 1064 identified variants with at least four allele counts. Genes considered include CDKN2A, CDKN2B, and noncoding transcript ANRIL (CDKN2BAS1). (C) List of top 10 longevity‐associated variants.

(A)				
MAF Range	SNP: *N*=2056		Complex: *N*=160	
	Known variants	Novel variants	Known variants	Novel variants
0%–0.5%	557	664	39	47
0.5%–1%	109	29	3	3
1%–5%	259	21	20	11
5%–10%	93	0	10	5
10%–25%	99	0	11	5
25%–50%	225	0	6	0

(B)					
	Total	SNP		Indels	
		Known Variants	Novel Variants	Known Variants	Novel Variants
Genic	765	597	106	36	26
Exonic	45	33	9	2	1
Synonymous	1	1	0	0	0
Nonsynonymous	7	4	3	0	0
Noncoding RNA	21	16	4	1	0
5' UTR	2	1	1	0	0
3' UTR	14	11	1	1	1
Intronic	720	564	97	34	25
Splicing	2	2	0	0	0
Intergenic	526	436	51	28	11

(C)						
Top longevity variants	Chr9 position (hg19)	Allele	MAF (case)	MAF (con)	*p* Value	Function
rs74605971	21,947,885	T > A	0.052	0.095	0.00044	Intergenic
rs12335941	21,955,669	G > A	0.339	0.413	0.00091	Intergenic
rs11521166	21,948,376	C > T	0.053	0.092	0.00147	Intergenic
rs111310495	21,990,187	A > G	0.002	0.016	0.00158	Intronic
Novel	22,068,072	G > A	0.002	0.016	0.00158	ncRNA_intronic
rs10757261	21,954,953	A > G	0.351	0.422	0.00159	Intergenic
rs2106118	21,949,528	T > A	0.401	0.473	0.00165	Intergenic
rs2106117	21,949,527	T > G	0.402	0.472	0.00226	Intergenic
rs10738612	22,174,103	T > C	0.438	0.506	0.00323	Intergenic
rs2518722	21,952,926	C > T	0.244	0.304	0.00398	Intergenic

We functionally annotated 1291 variants with at least four counts of minor alleles (Table 1b). The vast majority of these variants were either intronic (720, 55.8%) or intergenic (526, 40.7%). Among the 45 (3.5%) exonic variants, 21 were located in ANRIL and 10 in 5′ or 3′ UTR. We identified seven non‐synonymous and one synonymous SNPs, including three novel variants: one in the CDKN2A gene (chr9:21974675 A > C, V51G, MAF = 0.84%) and two in CDKN2B (chr9:22006101 C > T, R101Q, MAF = 0.21% and chr9:22008790 C > A, G55W, MAF = 0.26%). These candidate functional variants were not found to be associated with longevity (*p* > 0.05).

We identified 84 variants associated with longevity based on nominal *p*‐values (Table 1c). The majority of top hit SNPs were situated downstream of CDKN2A (Figure 1a). Among the GWAS‐reported SNPs, the variant with the highest significance was rs4977756 (*p* = 0.019, OR = 0.78), which has been linked to glaucoma (Burdon et al., 2011). This variant was also reported in a previous longevity iGWAS study using the same cohort (Fortney et al., 2015). Most GWAS variants were not found to be significantly associated with longevity in this study, including rs1333049 (*p* = 0.36, OR = 0.92), the strongest coronary artery disease variant (Table S1). However, a lower odds ratio for the risk allele was consistently observed for centenarians across all but one trait, suggesting a trend of combined risk variant depletion (Figure 1b). The only exception was glioma (rs1412829), which could be attributed to its non‐risk allele being linked to the risks of other diseases, such as glaucoma and cardiovascular traits.

**FIGURE 1:**
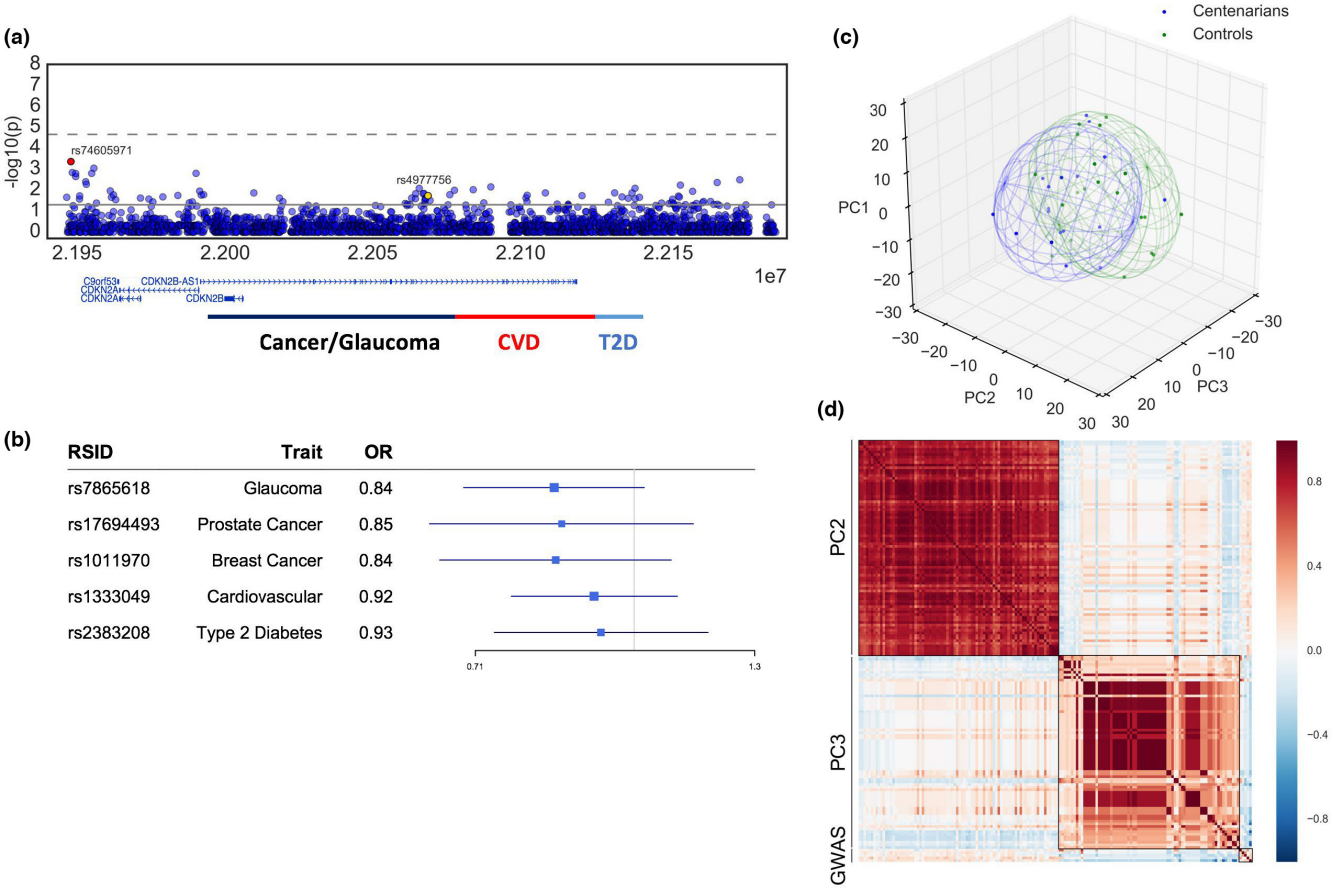
(a) Regional plots of 9p21.3 genotype‐longevity associations. Solid line and dash line represent nominal *p* and adjusted p threshold, respectively. Subdivisions of the locus with enriched GWAS variants are indicated at bottom. (b) Forest plot for five distinct GWAS SNPs representing major disease traits associated with the 9p21 locus (c) Distribution of first three principal components from PCA for the 18 centenarian pools and 20 control pools. Ellipsoids indicate distribution confidence interval. (d) Correlation matrix between the high importance SNPs from PC2 and PC3 and the GWAS risk variants in (B).

We probed whether the longevity variants downstream of CDKN2A and within the GWAS region are associated with distinct longevity signals, or co‐occur due to linkage disequilibrium (LD). Our correlation analysis of the pools shows that the SNPs cluster into five groups with minimal interdependency, aligning with the five major LD blocks in the sequenced region (Figure S2). To tackle multicollinearity (Chowdhury et al., 2021), we used nonparametric machine learning, specifically random forest and boosting techniques (Ogutu et al., 2011). From this, we identified high feature importance variants at both CDKN2A and rs4977756 sites (Figure S3). These combined results suggest the longevity association of 9p21.3 is likely polygenic.

To statistically test the significance of GWAS risk allele depletion in centenarians, we reduced the complete list of GWAS variants to five uncorrelated (*r* ^2^ ≤ 0.1) tag SNPs (Figure S4) (Machiela & Chanock, 2015). Each tag SNP represented its linked variants, typically associated with the same traits, and we examined their combined distribution using a permutation test. The result was significant (*p* < 10^−4^), suggesting that the overall age‐related disease risks in the 9p21.3 region were lower in centenarians.

Meanwhile, principal component analysis (PCA) revealed major differences of the 9p21.3 genotype in control and longevity cohorts within the first three principal components (Figure 1c). Further analysis of individual principal components (PCs) showed that the centenarian group had notably lower PC2 (*p* = 0.036, Mann–Whitney *U*‐test) and higher PC3 (*p* = 0.089) values. Applying a support vector classifier (SVC), we found the two PCs significantly distinguished the two cohorts (accuracy = 74%, *p* = 0.017, permutation test).

To affirm the significant differences in principal components (PCs) between the two groups, we evaluated the potential for technical confounders such as batch effects. This was done by analyzing the 34,954 variants on 360 targeted gene exon regions sequenced alongside the 9p21.3 from the same cohort (Ryu et al., 2018). The exon data showed high uniformity among pools (Figure S5A), and the first three PCs did not exhibit significant case versus control difference (Figure S5B,C), suggesting that the separation is exclusively associated with the 9p21.3 genotypes.

Upon examining the feature importance in these components, we discovered that PC2 and PC3 were characterized by two distinct groups of linked low‐frequency common variants (MAF <5% for both PC2 and PC3) (Figures S6 and S7A). Interestingly, the minor alleles of variants with high importance in PC2 were associated with the risk alleles of all five flag GWAS SNPs, while those in PC3 were linked to the non‐risk alleles (Figure 1d). This finding suggests that PC2 and PC3 represent low‐frequency genotypes associated with high and low combinatorial disease risks, respectively. Hence, the low PC2 and high PC3 scores in centenarians indicate an enrichment of genotypes with overall reduced genetic risks for age‐related diseases in the 9p21.3 region, which aligns with the analysis of GWAS SNPs.

To assess the association of clustered rare variants with longevity, we performed sequence kernel association test (SKAT) (Wu et al., 2011). After breaking down the locus by position of the genes and performed SKAT separately, the association of CDKN2A downstream region was found significant when the direction of variants was considered (Table S3). We also performed SKAT on potential regulatory elements in 127 epigenomes from Roadmap Project. No significant association was identified (data not shown). Nevertheless, the minor allele of the majority of variants in this region, both rare and common, was depleted in centenarians, indicating a possible deleterious role of alternative alleles in longevity for variants in this region (Figure S7B).

In this study, we conducted a comprehensive sequencing analysis of the 9p21.3 locus, which is associated with multiple age‐related phenotypes, in Ashkenazi Jewish centenarians. To our knowledge, this study is the first to extensively characterize the association of all genetic variants in this locus with extreme longevity in a significant cohort size. We identified moderate associations between multiple GWAS risk variants in 9p21.3 and longevity, with the strongest signal originating from rs4977756, a variant reported to be associated with glaucoma risks (Burdon et al., 2011). Notably, rs4977756 is in high LD with CAD variants (*R* ^2^ = 0.41 in Europeans) and located at the junction between cancer/glaucoma and CAD blocks. This LD block junction region represents the strongest longevity hotspot within the 9p21.3 GWAS locus (Figure 1a). Together with the result that the depletion of risk alleles was moderate but consistent for all age‐related disorder variants, our data suggest that instead of potently evading the risk of one particular trait associated with 9p21.3, the Ashkenazi Jewish centenarians may carry an overall lower genetic risk at this locus.

Consistent with our findings from single‐variant analysis, we identified two distinct variant groups that are either enriched or depleted in centenarians. Despite their low minor allele frequencies, both variant groups showed strong correlations in sample distribution (Figure 1d). Although further haplotyping is required for confirmation, such patterns strongly suggest that the minor alleles of these variants belong to one or a few haplotypes. We demonstrated that these two variant groups are associated with combined GWAS non‐risk and risk alleles, which are enriched and depleted in centenarians, respectively. These findings indicate the presence of rare high and low combined disease risk haplotypes in the 9p21.3 region, which are respectively negatively and positively selected in centenarians. To validate the presence and heritability of the protective haplotypes in centenarians, future studies should be performed to identify these haplotypes in their offspring.

Despite being one of the earliest identified GWAS loci, the mechanism by which 9p21.3 contributes to disease risk remains largely unclear. It has been demonstrated that the noncoding variants within this locus have regulatory functions and alter the expression levels of neighboring genes, including INK4/ARF and the long noncoding RNA transcript CDKN2B‐AS1 (Almontashiri et al., 2015; Harismendy et al., 2011). By sequencing the 9p21.3 locus, we provide a comprehensive list of variants associated with longevity, which serves as a valuable resource for further study of the regulatory mechanisms of this locus.

## AUTHOR CONTRIBUTIONS

Conceptualization: YZ, YS; methodology: YZ, SR, AT; analysis: YZ; resource: GA, NB; drafting: YZ, YS; editing: all authors.

## FUNDING INFORMATION

Research in the Suh lab was supported by the National Institute of Health (AG017242, DK127778, AG076040, AG069750, AG061521, GM104459, AG056278, AG057341, AG057433, AG057706), a grant GCRLE‐1320 from the Global Consortium for Reproductive Longevity and Equality at the Buck Institute, made possible by the Bia‐Echo Foundation, and a grant from The Simons Foundation.

## CONFLICT OF INTEREST STATEMENT

The authors declare no conflict of interest.

## Supporting information


Appendix S1

Table S2

## Data Availability

Data available on request from the authors.
