# Supplementary material for: Targeted sequencing of the 9p21.3 region reveals association with reduced disease risks in Ashkenazi Jewish centenarians
Source: Aging Cell. 2023 Aug 22;22(10):e13962. doi: 10.1111/acel.13962 (PMC10577543; doi:10.1111/acel.13962)
Supplement: Supplementary file 1 — Appendix S1 [file ACEL-22-e13962-s002.pdf]

Experimental Procedures

Subjects

The Ashkenazi Centenarian cohort has been described in our previous studies (Shlush et al. 2008; Ryu et al. 2016). Briefly, 450 centenarians (mean age: 98) and 500 controls (mean age: 73) with no family record of longevity were involved in the study. Genomic DNA was extracted from the participants' whole blood samples of participants and subjected to whole genome amplification using GenomiPhi V2 DNA Amplification kits (GE Healthcare Life Sciences) prior to capture genome sequencing.

Pooled Target Capture Sequencing

The pooled capture-seq technique was performed as described previously (Ryu et al. 2018). In short, genomic DNA samples were divided into groups of 25 and pooled equimolarly. This resulted in 18 pools of centenarians and 20 pools of controls. Using one microgram of pooled DNA, the sequencing library was prepared following Illumina's Truseq library protocol. The 9p21.3 region (chr9:21950000-22180000, hg19) was specifically captured using the Nimblegen SeqCap EZ Choice library target capture kit according to the manufacturer's instructions. The capture and library preparation procedures were performed in a single batch experiment for all 38 pools. Sequencing was performed on the Illumina HiSeq2500 platform with an average depth of 30x per individual. Sequencing results were aligned to the human genome hg19 using BWA. After pre-processing with PICARD tools (http://broadinstitute.github.io/picard), multi-sample variant calling was achieved using CRISP (Bansal 2010). The variants were annotated to dbSNP149 and refGene for functional annotation/prediction. ANNOVAR was used for functional annotation (Wang et al. 2010).

Genotyping

Genomic regions with variants subject to genotyping were PCR amplified from genomic DNA using FastStart Taq DNA polymerase (Roche). Genotyping was performed on the Sequenom MassARRAY platform following the manufacturer's instructions. Genotype calls were performed using TYPER 4.0, the coupled analysis software for the assay, with default call thresholds.

Statistical Analysis

Allele frequencies for each variant in the centenarian and control groups were calculated, and statistical inference of association was determined using Fisher's exact tests. For rare variant analysis, the Sequence Kernel Association Test (SKAT) (Wu et al. 2011) and its derivative methods (SKAT-O, SKAT-C) were applied to the entire sequenced interval, as well as subdivisions based on gene locations. As previously described (Ryu et al. 2021), analysis was performed by using minor allele frequency (MAF) of variants in each pool instead of allele counts in each

individual. Reported p-values were adjusted for multiple testing using the Benjamini-Hochberg procedure. For permutation tests for combined depletion of risk variants, identification tags for pools were permuted 10,000 times. For each permuted dataset, the Z-scores of the five GWAS SNPs shown in Figure 1B were calculated for the centenarian group. The number of incidences in which the sum of Z-scores was equal to or less than that of the actual centenarian group were counted and then divided by the total number of trials to calculate p-values.

The Python Sklearn package was utilized for the feature selection study. Before initiating the learning process, the allele frequency matrix was pre-processed by 1) adjusting the allele count to the minor allele (for cases where the major allele was the alternative), 2) removing singletons and doubletons, and 3) normalizing to Z-score. Random Forest and Gradient Tree Boosting classifiers were implemented using the RandomForestClassifier (n_estimator=10000) and GradientBoostingClassifier (n_estimator=5000, learning_rate=0.02) in the ensemble module, respectively. Principal Component Analysis (PCA) was performed using the PCA function in the decomposition module. A linear Support Vector Classifier (SVC) was built to separate control and centenarian pools using the first 3 PC values with the SVC function in the svm module. The significance of separation was tested by permuting the pool's identification tags 10,000 times and counting instances where classification accuracy was equal to or higher than the actual data. The python seaborn.heatmap function was used to generate a correlation matrix heatmap. A threshold of 0.05 was applied for PC2 and PC3 to select the important feature SNPs.

Data Usage

The Linkage Disequilibrium (LD) information for the European population (CEU) was obtained from the 1000 Genomes Project. The LD matrices for selected variants in Figures S2 and S4 were plotted using the online LDlink tool (Machiela and Chanock 2015). The same-batch exon sequencing results for the Ashkenazi Jewish cohort were obtained from the authors of a previous study (Ryu et al. 2021).

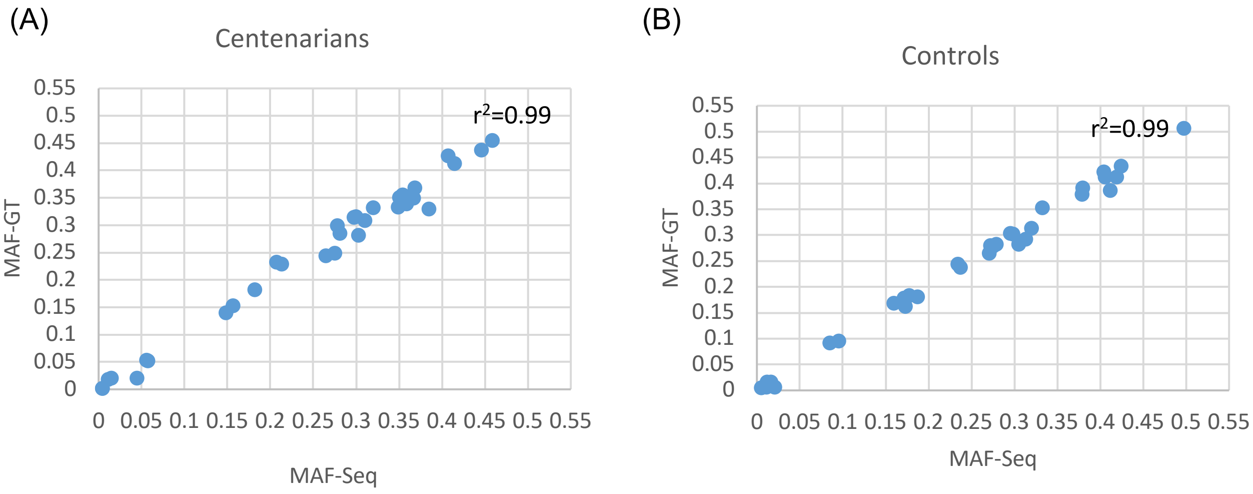

Figure S1 Genotyping validation of variant allele frequency called from pooled capture sequencing for (A) centenarians and (B) controls. See Table S1 for list of assayed variants.

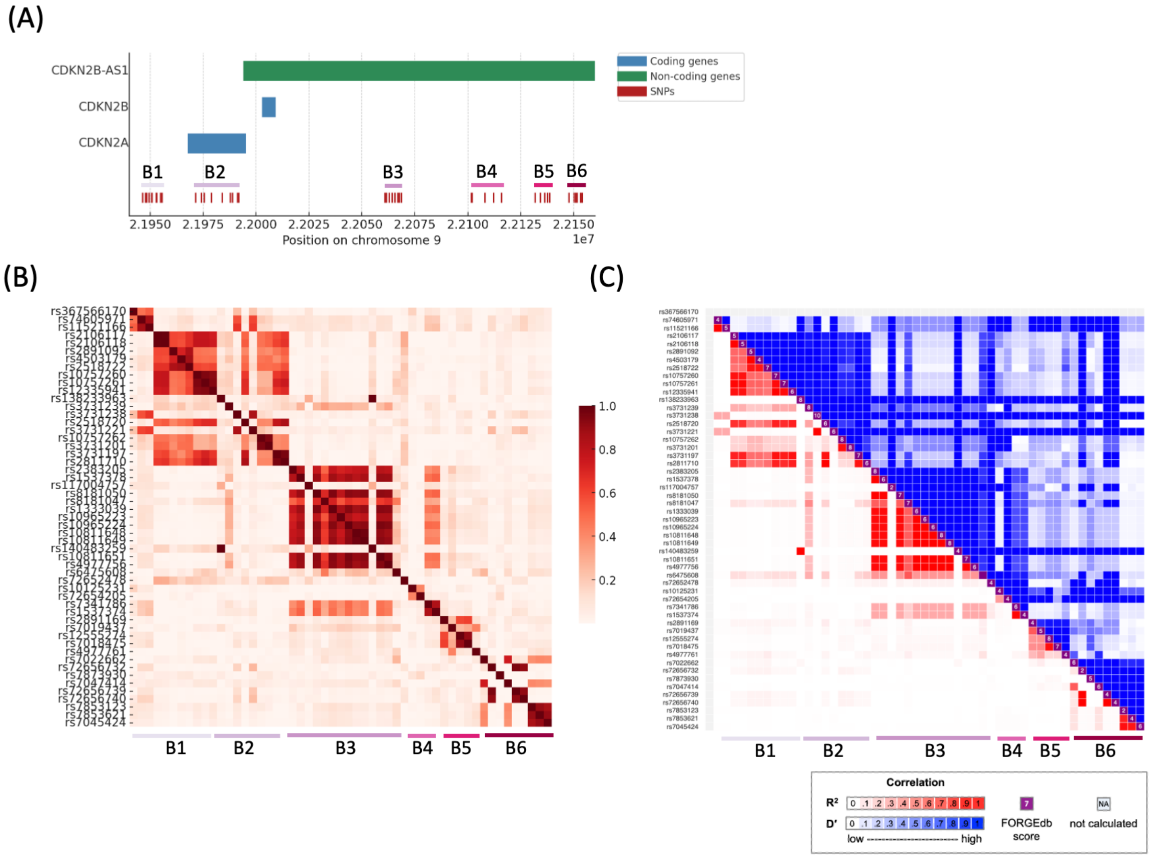

Figure S2 (A) Distribution of variants with nominally significant ($p < 0.05$) difference in MAFs between centenarians and controls in 9p21.3 region. (B) Pearson correlation (R2) for the allele frequency of significant in all 38 sequencing pools. (C) Linkage disequilibrium map of these variants in European (CEU) population.

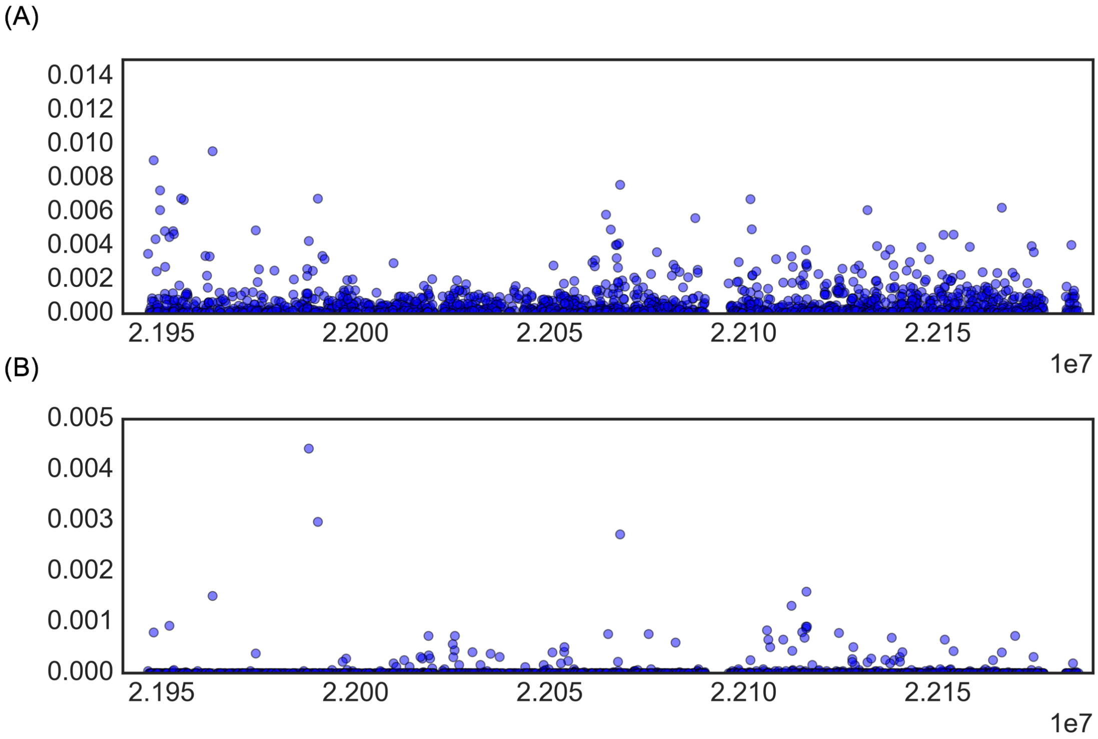

Figure S3 Feature importance for all variants in (A) random forest classifier and (B) stochastic gradient boosting classifier for centenarian association.

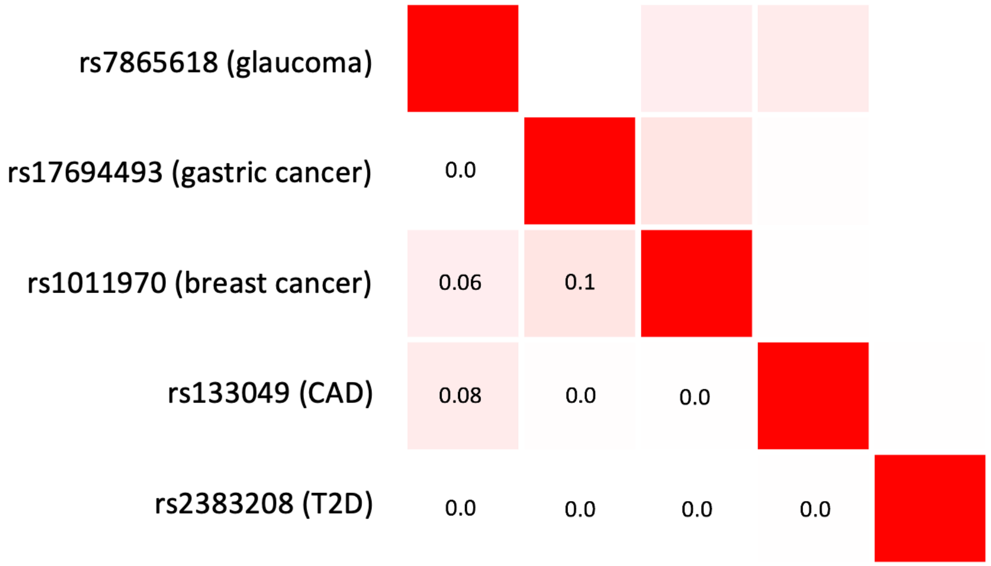

Figure S4 LD matrix for the five representative GWAS SNPs in all populations from LDLink database. Numbers in the squares indicate R squared correlation.

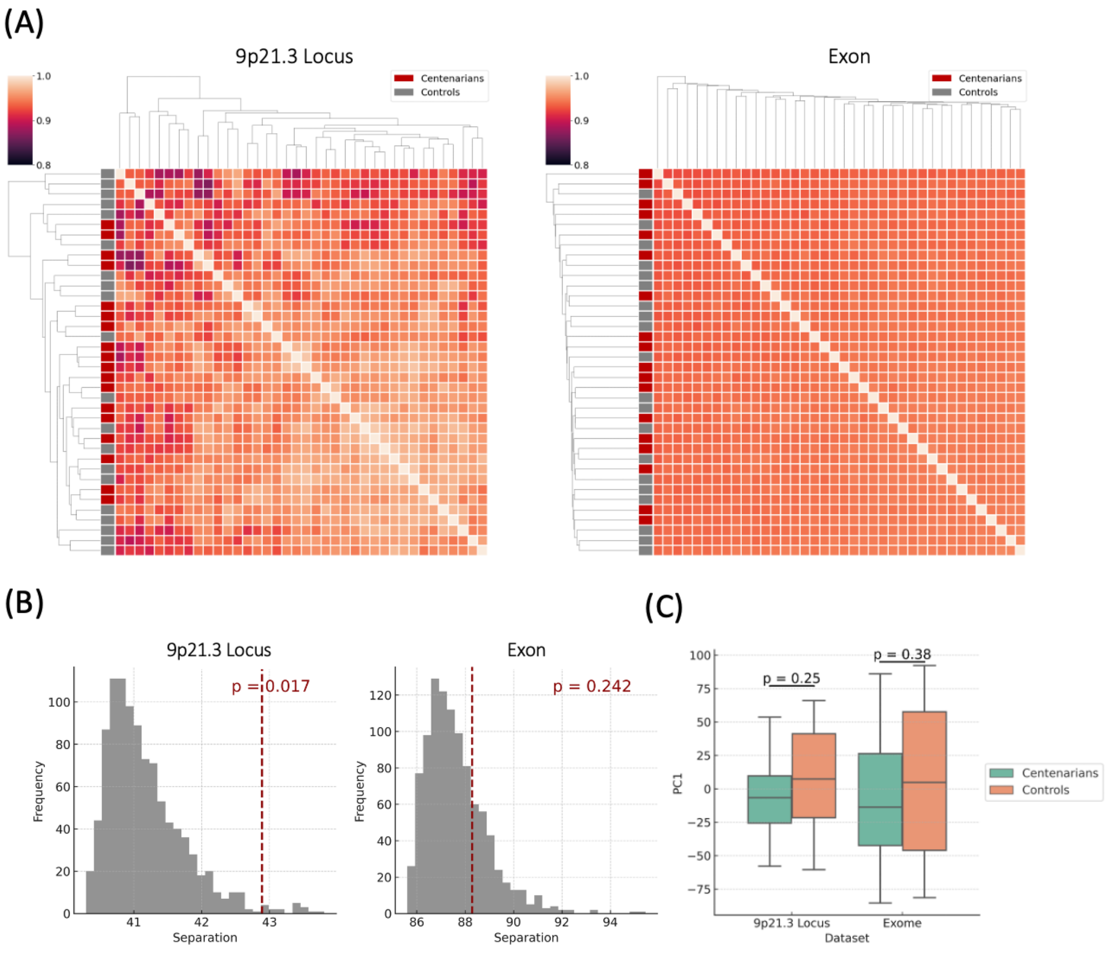

Figure S5 Comparison of the result between 2,216 9p21.3 variants in this study and 34,954 variants on 360 gene exons that were sequenced together in the same batch. (A) Cluster map of the sample pools by Pearson correlation. (B) Permutation test of Euclidian distance separation between control and centenarian groups by the PC2 and PC3 from the PCA analysis of the two datasets. (C) Boxplot showing the distribution of PC1 in control and centenarian groups. Significance of the difference was calculated by Mann-Whitney U test.

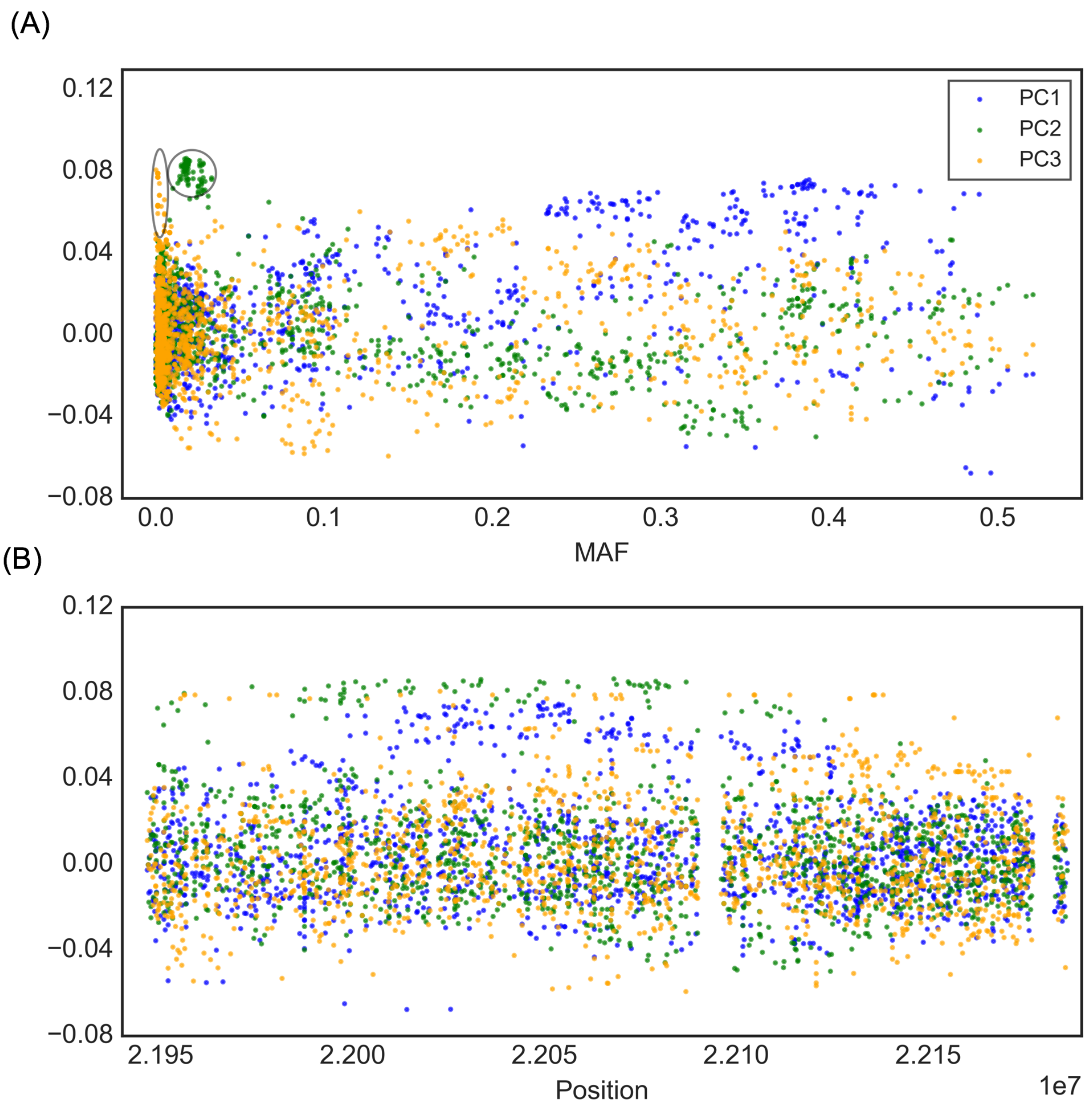

Figure S6 2D plots denoting feature weight (y xias) for all SNPs in the first 3 principle components versus their (A) minor allele frequency and (B) position.

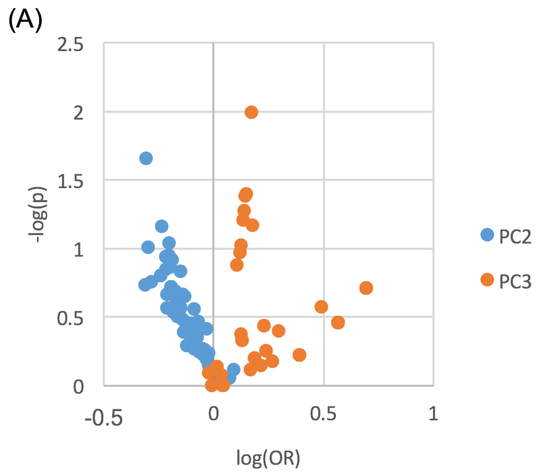

Figure S7 Volcano plots for (A) important feature SNPs in the principle components 2 and 3 and (B) CDKN2A-downstream SNPs.

	MAF-Sequecing		MAF-Genotyping	
ID	Centenarians	Controls	Centenarians	Controls
rs111310495	0.002	0.016	0.004	0.016
Nov22068072	0.002	0.016	0.004	0.012
Nov22089832	0.020	0.006	0.045	0.021
rs576966099	0.018	0.005	0.011	0.005
rs1011970	0.140	0.162	0.148	0.173
rs1063192	0.309	0.283	0.310	0.279
rs10738612	0.438	0.506	0.446	0.497
rs10757260	0.350	0.413	0.367	0.419
rs10757261	0.351	0.422	0.351	0.404
rs10757272	0.413	0.379	0.415	0.379
rs10811661	0.182	0.169	0.182	0.160
rs11515	0.153	0.183	0.157	0.178
rs11521166	0.053	0.092	0.056	0.085
rs12335941	0.339	0.413	0.359	0.406
rs1333039	0.299	0.244	0.278	0.235
rs1333040	0.332	0.302	0.320	0.299
rs1333042	0.369	0.353	0.369	0.333
rs1333049	0.456	0.434	0.459	0.424
rs1412829	0.281	0.265	0.303	0.270
rs1412832	0.229	0.181	0.213	0.187
rs2157719	0.314	0.280	0.298	0.272
rs2383207	0.356	0.313	0.355	0.320
rs2518722	0.244	0.304	0.265	0.295
rs3731197	0.330	0.386	0.385	0.412
rs3731211	0.249	0.282	0.275	0.305
rs4977574	0.427	0.392	0.407	0.379
rs4977756	0.286	0.238	0.281	0.237
rs523096	0.333	0.293	0.350	0.314
rs72652408	0.021	0.007	0.015	0.011
rs74605971	0.052	0.095	0.057	0.095
rs75059952	0.232	0.179	0.207	0.172
rs7865618	0.316	0.279	0.300	0.275

Table S1: List of variants for validation analysis by genotyping.

Table S2 List of variants validated by genotyping (attached xlsx separately)

	SKAT-O	SKAT	SKAT-C
CDKN2A_downstream	0.0024*	0.1380	0.0282*
CDKN2A	0.7045	0.4285	0.1655
CDKN2B	1.0000	0.8812	0.4409
CDKN2BAS1	1.0000	0.8812	0.4250
CDKN2BAS1_downstream	1.0000	0.8812	0.4212
Total	0.9904	0.8812	0.3128

Table S3: Adjusted p value from SKAT analysis of sequenced region and subdivisions.

Reference

Bansal V. 2010. A statistical method for the detection of variants from next generation resequencing of DNA pools. *Bioinformatics* **26**: i318-324.

Machiela MJ, Chanock SJ. 2015. LDlink: a web-based application for exploring population-specific haplotype structure and linking correlated alleles of possible functional variants. *Bioinformatics* **31**: 3555-3557.

Ryu S, Atzmon G, Barzilai N, Raghavachari N, Suh Y. 2016. Genetic landscape of APOE in human longevity revealed by high-throughput sequencing. *Mech Ageing Dev* **155**: 7-9.

Ryu S, Han J, Norden-Krichmar TM, Schork NJ, Suh Y. 2018. Effective discovery of rare variants by pooled target capture sequencing: A comparative analysis with individually indexed target capture sequencing. *Mutat Res* **809**: 24-31.

Ryu S, Han J, Norden-Krichmar TM, Zhang Q, Lee S, Zhang Z, Atzmon G, Niedernhofer LJ, Robbins PD, Barzilai N et al. 2021. Genetic signature of human longevity in PKC and NF-kappaB signaling. *Aging Cell* **20**: e13362.

Shlush LI, Atzmon G, Weisshof R, Behar D, Yudkovsky G, Barzilai N, Skorecki K. 2008. Ashkenazi Jewish centenarians do not demonstrate enrichment in mitochondrial haplogroup J. *PLoS One* **3**: e3425.

Wang K, Li M, Hakonarson H. 2010. ANNOVAR: functional annotation of genetic variants from high-throughput sequencing data. *Nucleic Acids Res* **38**: e164.

Wu MC, Lee S, Cai T, Li Y, Boehnke M, Lin X. 2011. Rare-variant association testing for sequencing data with the sequence kernel association test. *Am J Hum Genet* **89**: 82-93.
